# Radiographic Evaluation of the Surgical Treatment of Pediatric Supracondylar Humeral Fractures

**DOI:** 10.1055/s-0044-1787765

**Published:** 2024-08-01

**Authors:** Gabriel Rigatti, Sérgio Roberto Canarim Danesi, Rafaela Dias Barbosa, Douglas Backes Schreiner

**Affiliations:** 1 Departamento de Ortopedia e Traumatologia, Hospital Cristo Redentor, Grupo Hospitalar Conceição, Porto Alegre, RS, Brasil; 2 Ortopedia Pediátrica, Hospital Cristo Redentor, Grupo Hospitalar Conceição, Porto Alegre, RS, Brasil; 3 Cirurgia de Ombro e Cotovelo, Santa Casa de Misericórdia de Porto Alegre, RS, Brasil

**Keywords:** child, elbow, fracture fixation, internal, humeral fractures

## Abstract

**Objective** To perform a radiographic assessment of the quality of supracondylar fracture fixation by identifying the factors that have contributed to inadequate reduction and increased the chance of reduction loss during outpatient follow-up. The variables analyzed were as follows: fracture line, initial displacement, time of day the surgery was performed, and chosen fixation technique.

**Methods** Review of electronic medical records and radiographic evaluation of supracondylar fractures operated from January 2017 to December 2022. The radiograph assessment was based on the Baumann angle and the anterior humeral line. Determination of fixation quality was based on the number of cortices, crossing site, and wire divergence.

**Results** We evaluated 194 cases, and postoperative reduction was poor in 17% of the subjects. Reduction loss occurred in 39 cases (20.10%), and 19 (48.7%) of these patients presented insufficient fixation ( *p* = 0.002). Among the cases operated during the day, 12.5% lost the reduction compared with 32% of the patients who underwent surgery at night and early in the morning ( *p* = 0.001).

**Conclusion** Reduction quality and postoperative fixation loss were closely related to technical errors and the time of day the surgery was performed.

## Introduction

Supracondylar humerus fractures account for up to 15% of all childhood fractures and for 60% of all pediatric elbow fractures. 1 2 The maximum incidence occurs from 5 to 7 years of age, peaking at age 6. 3 4 Supracondylar humerus fractures are those that most frequently require surgical treatment in the pediatric population. 5

The most common trauma mechanism is fall with a flat hand and hyperextension of the elbow, resulting in a fracture extending to the distal fragment. The rarer flexion type results from direct trauma to the posterior region of the elbow, leading to an anterior displacement of the distal fragment. 6

Some variables can influence the quality of surgical reduction and contribute to the loss of postoperative fixation. The fracture line, the initial displacement, the time of day the surgery is performed, and the chosen fixation technique may directly relate to treatment success. The surgeon must understand the role of each variable and develop methods for better management. The present study aimed to map and evaluate the factors potentially impairing the quality of surgical reduction and contributing to postoperative fixation loss.

## Materials and Methods

The institutional Human Research Ethics Committee approved the present study under Opinion Certificate number CAAE 69485623.5.0000.5530.

We cross-sectionally and retrospectively evaluated all pediatric patients aged 1 to 14 years with supracondylar humeral fractures undergoing surgical treatment from January 1st, 2017, to December 31, 2022. We excluded patients with no postoperative follow-up, inadequate medical records, and incomplete radiographic study, in addition to those with fractures showing intra-articular extension.

In total, we analyzed 210 cases operated on by different surgeons. After applying the exclusion criteria, we included 194 patients in the study. We reviewed electronic medical records and radiographs, in addition to age, gender, laterality, fracture type (extension or flexion), line morphology, shift during surgery performance, and the presence of neurovascular complications. We classified fracture patterns using preoperative and intraoperative images according to the criteria described by Bakh et al. 7 ( Table 1 ).

**Table 1:** Fracture pattern, radiological representation, and trace stability

Fracture type	Image	Number of cases per fracture pattern	Instability	Definition
**Low transversal + low line in the sagittal plane**		131	Stable	< 10° obliquity in the coronal plane with transverse fracture close to the epicondyles + < 20° inclination in the sagittal plane
**Low transversal + high line in the sagittal plane**		42	Unstable	< 10° obliquity in the coronal plane with transverse fracture close to the epicondyles + > 20° inclination in the sagittal plane
**Lateral coronal oblique**		6	Unstable	≥ 10° obliquity in the coronal plane with fracture line laterally higher
**Medial coronal oblique**		7	Unstable	≥ 10° obliquity in the coronal plane with fracture line medially higher
**High coronal**		8	Unstable	Fracture with a line above the olecranon fossa, but within the distal metaphysis of the humerus

**Note:** Table with changes based on Bahk et al. (2008). 7

**Fig. 1:**
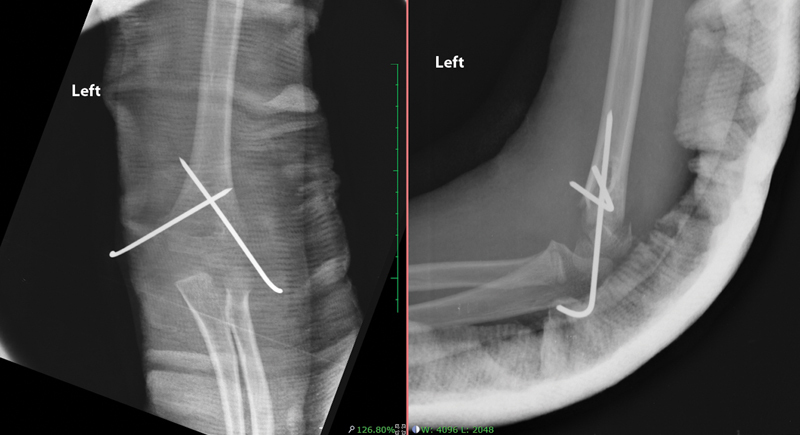
Anteroposterior (AP) and lateral radiographs of the distal humerus performed immediately after surgery, demonstrating unsatisfactory reduction, as the anterior humeral line does not touch the capitellum. In the AP radiograph, the ulnar spine wire does not appear to fixate the distal fragment.

We assessed the quality of postoperative reduction in the coronal plane using the Baumann angle (normal range: 9–26° 8 ) and in the sagittal plane using the anterior humeral line (which is normal when crossing the central and anterior thirds of the capitellum). The reduction was inadequate when the Baumann angle was outside the normal criteria, the anterior humeral line did not pass through the capitellum, or both ( Fig. 1 ). The reduction loss criteria during follow-up were a 6° change in the Baumann angle 9 or alterations in the thirds of the capitellum's intersection with the anterior humeral line on lateral radiographs.

**Fig. 2:**
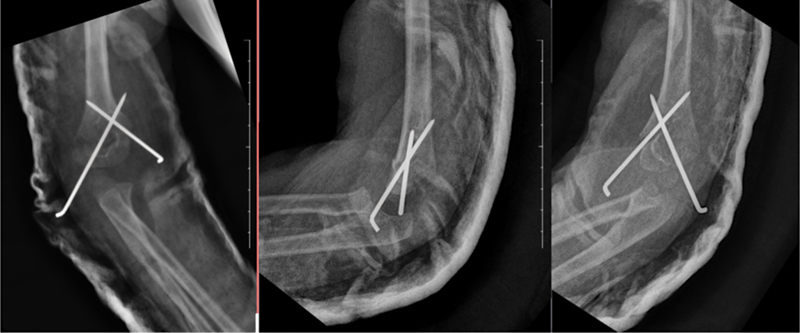
Anteroposterior and lateral oblique radiographs of the distal humerus performed immediately after surgery, demonstrating an acceptable reduction but inadequate fixation, as one of the wires does not fixate the distal fragment.

We also assessed the variables regarding the configuration of the fixation, such as the order of the wires and the stability promoted (number of cortices, crossing site, wire divergence). The fixation technique was inadequate if presenting: 1) a lack of cortical fixation of the distal or proximal fragment by 1 or more wires, totaling less than 4 cortical wires fixed; 2) wire convergence in the proximal cortex; or 3) pins crossing in the fracture focus 10 ( Fig. 2 ). In the present study we considered the fixation good when it included the four cortices, two in the proximal fragment and two in the distal fragment, with adequate separation to fix the two columns. We stratified the time of day for surgery performance into daytime (7 am–7 pm), nighttime (7 pm–midnight), or early morning (midnight–7 am).

Data were collected through a review of the medical records and assessed using specific statistical tests (the Chi-squared test) in the application of the IBM SPSS Statistics for Windows (IBM Corp., Armonk, NY, United States) software, version 25.0, with a significance level of 0.05. Project approval followed Resolution no. 466/2012 of the Brazilian National Health Council (Conselho Nacional de Saúde, CNS, in Portuguese) and current complementary ethical regulations.

## Results

We evaluated 194 cases of supracondylar fractures undergoing surgical treatment in the estimated period. These sample included 133 male (68.6%) and 61 female patients (31.4%). The peak incidence ranged from 4 to 7 years of age, with a mean age of 6. The left side was the most affected, accounting for 57.8% of the cases. Regarding fracture displacement, 171 were in extension (87.6%), and 24, in flexion (12.4%).

Neurological injury was observed in 13 (6.7%) cases, with ulnar nerve involvement in 5 patients, median nerve injury in 4 patients, radial nerve injury in 3 patients, and radial nerve injury and impairment in 1 patient. All cases achieved complete recovery from neurological injury within 6 months. The most severe complication was compartment syndrome, which occurred in 1 case and required fasciotomy but progressed to Volkmann ischemic contracture. Regarding the approach, 4 cases (2.1%) underwent open reduction, while the others underwent indirect reduction and percutaneous fixation.

We also classified fractures per the fracture line: 173 fractures were low transversal, 7 were medial oblique, 6 were lateral oblique, and 8 were transversal high. Among the low transversal fractures, 42 cases presented a high line in the sagittal plane, leading to instability. We considered 63 fractures (32.47%) unstable due to their morphology.

Crossed wire fixation occurred in 125 cases (64.5%), using 2 lateral wires in 35 cases (18%), 2 lateral wires and 1 medial wire in 27 cases (14%), 3 lateral wires in 6 cases (3%), and atypical configuration with 1 lateral wire and 3 medial wires in 1 subject (0.5%). In total, 55 cases (28.35%) presented insufficient fixation.

Postoperative reduction was acceptable in 161 patients (82.9%). In 33 fractures (17%), the fixation occurred in non-ideal parameters regarding the Baumann angle and the anterior humeral line, and 5 underwent reintervention. The main indication of poor reduction occurred in the sagittal plane when the anterior humeral line did not meet the anterior third of the capitellum. Poorly-reduced fractures included a single case of Gartland type-II, while the remaining were type-III (p = 0.042). Regarding fracture line instability, among the 33 unstable fractures, 15 (45.45%) presented inadequate reduction in the immediate postoperative period (p = 0.105).

Regarding the time of surgery performance, 19 patients (25.7%) treated during the night or early morning shifts presented unsatisfactory reduction compared with 14 (11.7%) operated on during the day (p = 0.020).

**Fig. 3:**
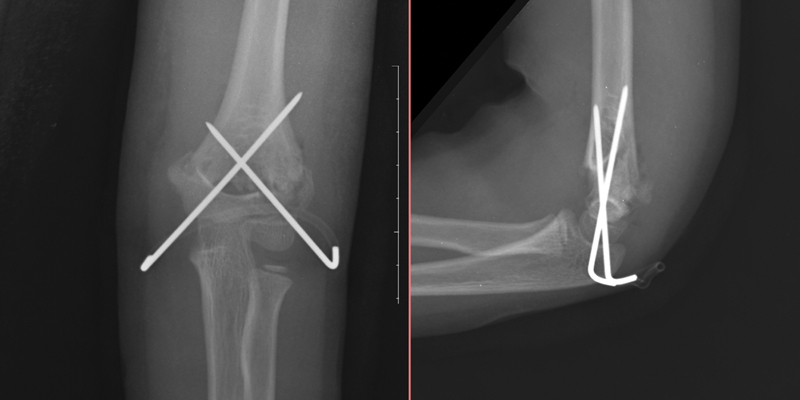
Anteroposterior and lateral radiographs of the distal humerus immediately after surgery.

**Fig. 4:**
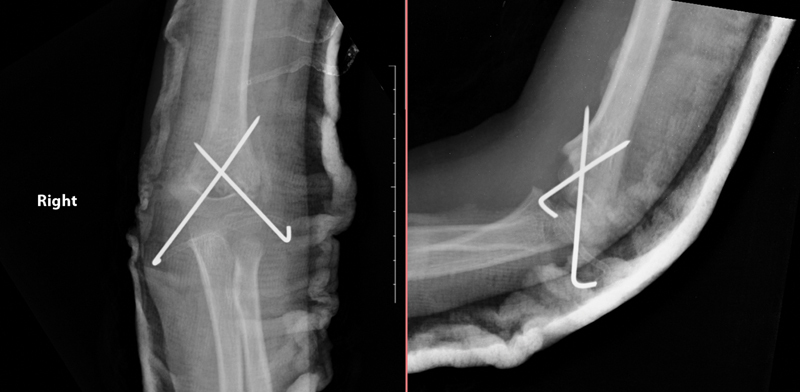
Anteroposterior and lateral radiographs of the same patient in fig. 3 three weeks after fixation, demonstrating reduction loss.

Reduction loss occurred in 39 cases (20.10%), including 37 Gartland type-III fractures (p = 0.032) and 16 (42%) morphologically-unstable fractures (p = 0.174). Reduction losses occurred in the coronal plane (Baumann angle) in 24 patients, in the sagittal plane (anterior humeral line) in 8 subjects, and in both planes in 7 cases ( Figs. 3 and 4 ). Of all patients with reduction loss, 19 (48.7%) presented insufficient fixation (p = 0.002). Reduction loss occurred in 15 cases (12.5%) operated on during the day and in 24 (32%) subjects operated on at night or early in the morning (p = 0.001).

## Discussion

The epidemiology regarding the age of the patients with supracondylar fractures of the humerus is consistent with most references, and these injuries are more frequent in patients aged 4 to 7 years. 3 4 Although fractures in extension prevailed in the sample of the present study, there was a high incidence of deviations in flexion (12.4%) compared with that of the world literature (2%). 8

Isolated neuropraxia of the anterior interosseous nerve (AIN) is the most common nerve injury in extension deviations, 11 while ulnar nerve compromise occurs in 90% of flexion fractures. 12 It is estimated that iatrogenic ulnar injury occurs in 1 in every 28 patients (4%) undergoing cruciate fixation. 13 Therefore, the higher incidence of 12.4% of ulnar involvement in the sample of the present study may be related to the increased incidence of fractures in flexion and the widespread use of cruciate fixation. The most severe complication was compartment syndrome, which occurred in a single case (0.51%), which is consistent with the study by Omid et al., 4 who reported an incidence ranging from 0.1 to 0.5%. The low incidence of complications related to the time between injury and fixation corroborates the planned and safe surgical management without the need for an urgent-emergency approach in most cases.

Most fracture treatments involve indirect reduction and percutaneous fixation. The need for open reduction is rare; it occurs in around 6% of the cases. 8 In the present study, open reduction was used 4 times (2.1%). The main indications for open reduction include irreducible, open fractures, or decreased perfusion after reduction. 14

The rate of reduction loss after supracondylar fracture fixation ranges from 1.6 to 33.3%. 15 According to Skaggs and Flynn, 14 the main cause for this loss is inadequate fixation attributed to technical errors. The lateral entry pins must be divergent, seeking maximum spacing at the fracture focus. 16 They must not converge or cross at the fracture focus; bicortical fixation is essential. 11 In the present study, insufficient fixation occurred in 48.7% of the cases of reduction loss ( *p* = 0.002), being a fundamental causal factor for unsatisfactory outcomes. The high heterogeneity of surgeons on duty and the fact that is the procedures were performed at a training service for new traumatologists are relevant factors in this context.

The time of surgery performance proved to be a critical variable for the radiological outcome: 32% of the cases operated on at night or early in the morning had unsatisfactory radiographs at the end of the follow-up period. The search for a causal justification for this data involves the fact that the surgeons are outside their most comfortable context, that is, on the third shift and with a reduced assistant team. The technical capacity or training of the surgeon should not be a determining factor, since night surgeries are performed by the same group of surgeons as daytime surgeries. This fact cannot be attributed exclusively to the orthopedic surgeon, as approximately 30 on-call staff perform day and night shifts and the same surgery at different times regardless of their subspecialty. Surgical treatment at night was associated with a higher rate of inadequate fixation compared with daytime procedures. 17 Delaying surgery until the following day does not increase the number of complications, as long as warning signs, such as exposure, vascular injury, and secondary complex signs (voluminous edema, ecchymosis, soft tissue pinching), are absent. 18

Some limitations to this analysis require consideration, including the retrospective nature and data collection according to available medical records. Furthermore, it is essential to further study the clinical correlation of the radiological findings herein described.

## Conclusion

The quality of the surgical reduction and its maintenance in the postoperative follow-up were closely related to technical aspects and the time of day of the surgery was performed. Therefore, except for specific situations of greater risk, delaying surgery until the following day results in a better radiological outcome without increasing the incidence of complications.
