# Supplementary material for: Radiographic Evaluation of the Surgical Treatment of Pediatric Supracondylar Humeral Fractures
Source: Rev Bras Ortop (Sao Paulo). 2024 Aug 1;59(4):e607–12. doi: 10.1055/s-0044-1787765 (PMC11374409; doi:10.1055/s-0044-1787765)
Supplement: Related texts

# Research article: Avaliação radiográfica do tratamento cirúrgico das fraturas supracondilianas do úmero em crianças

**Keywords:** cotovelo, criança, fixação interna de fraturas, fraturas do úmero

## Abstract

**Objetivo** Avaliar de forma radiográfica a qualidade da fixação das fraturas supracondilianas, identificando os fatores que contribuíram para uma redução inadequada e que aumentaram a chance de perda de redução no seguimento ambulatorial. As variáveis analisadas foram: traço de fratura, deslocamento inicial, período do dia em que a cirurgia foi realizada e técnica de fixação escolhida.

**Métodos** Revisão de prontuário eletrônico e avaliação radiográfica das fraturas supracondilianas operadas de janeiro de 2017 a dezembro de 2022. A avaliação das radiografias foi baseada no ângulo de Baumann e na linha umeral anterior. Já a qualidade de fixação foi mensurada pelo número de corticais, pelo local de cruzamento e pela divergência dos fios.

**Resultados** Foram avaliados 194 casos, sendo a redução pós-operatória considerada ruim em 17%. A perda de redução aconteceu em 39 casos (20,10%), sendo que 19 (48,7%) desses pacientes apresentaram fixação insuficiente ( *p* = 0,002). Dos casos operados durante o dia, 12,5% perderam a redução, comparados a 32% dos realizados nos períodos noturno e da madrugada ( *p* = 0,001).

**Conclusão** Dessa forma, a qualidade da redução e a perda da fixação pós-operatória mostraram-se intimamente relacionadas aos erros técnicos e ao período do dia em que a cirurgia foi realizada.

## Introdução

As fraturas supracondilianas do úmero são responsáveis por até 15% de todas as fraturas na infância e por 60% de todas as fraturas do cotovelo em pacientes pediátricos. 1 2 A incidência máxima é na faixa etária de 5 a 7 anos, com pico aos 6 anos. 3 4 É a fratura na criança que mais frequentemente exige tratamento cirúrgico. 5

O mecanismo de trauma mais frequente é a queda com a mão espalmada e hiperextensão do cotovelo, que resulta em uma fratura com extensão do fragmento distal. O tipo em flexão, mais raro, é causado por trauma direto na região posterior do cotovelo, e resulta no deslocamento anterior do fragmento distal. 6

Há variáveis que podem influenciar na qualidade da redução cirúrgica e contribuir para a perda da fixação pós-operatória. O traço da fratura, o deslocamento inicial, o período do dia em que a cirurgia foi realizada e a técnica de fixação escolhida podem estar diretamente relacionados com o sucesso no tratamento. Entender o papel de cada variável e desenvolver métodos para conduzi-las melhor é papel do cirurgião. O objetivo deste estudo é mapear e avaliar os fatores que podem prejudicar a qualidade da redução cirúrgica e contribuir para a perda da fixação pós-operatória.

## Materiais e Métodos

Este estudo foi aprovado pelo Comitê de Ética em Pesquisa em Seres Humanos de nossa instituição sob o número do Certificado de Liberação CAAE: 69485623.5.0000.5530.

Avaliamos de maneira transversal e retrospectiva todos os pacientes pediátricos de 1 a 14 anos com fratura supracondiliana do úmero submetidos a tratamento cirúrgico 1° de janeiro de 2017 a 31 de dezembro de 2022. Os pacientes sem acompanhamento pós-operatório, sem registro adequado em prontuário, sem estudo radiográfico completo e com fraturas que apresentavam extensão intra-articular foram excluídos do estudo.

No total, foram analisados 210 casos operados por diferentes cirurgiões. Após a aplicação dos critérios de exclusão, 194 pacientes foram incluídos no estudo. Foram realizadas revisão de prontuário eletrônico e avaliação das radiografias. Dados relativos a idade, sexo, lateralidade, tipo de fratura (extensão ou flexão), morfologia do traço, turno de realização da cirurgia e presença de complicações neurovasculares foram analisados. Os padrões de fratura foram classificados por meio de imagens pré-operatórias e intraoperatórias de acordo com os critérios descritos por Bakh et al. 7 ( Tabela 1 ).

**Tabela 1:** Padrão de fratura, representação radiológica e estabilidade do traço

Tipo de fratura	Imagem	Número de casos de acordo com o padrão de fratura	Instabilidade	Definição
**Transversa baixa + traço baixo no plano sagital**		131	Estável	< 10° de obliquidade no plano coronal com fratura transversa próxima aos epicôndilos + inclinação < 20° no plano sagital
**Transversa baixa + traço alto no plano sagital**		42	Instável	< 10° de obliquidade no plano coronal com fratura transversa próxima aos epicôndilos + inclinação > 20° no plano sagital
**Coronal oblíqua lateral**		6	Instável	≥ 10° de obliquidade no plano coronal com traço de fratura mais alto lateralmente
**Coronal oblíqua medial**		7	Instável	≥ 10° de obliquidade no plano coronal com traço de fratura mais alto medialmente
**Coronal alta**		8	Instável	Fratura com traço acima da fossa do olécrano, mas dentro da metáfise distal do úmero

Nota: Tabela modificada com base em Bahk et al. (2008). 7

A qualidade de redução no pós-operatório foi avaliada no plano coronal pelo ângulo de Baumann (normalidade considerada de 9° a 26° 8 ), e, no plano sagital, pela linha umeral anterior (normalidade quando há cruzamento com os terços central e anterior do capítulo). A redução foi considerada inadequada quando o ângulo de Baumann estava fora dos critérios de normalidade e/ou a linha umeral anterior não passava pelo capítulo ( Fig. 1 ). Os critérios de perda da redução durante o seguimento foram definidos a partir de uma alteração de 6° no ângulo de Baumann 9 ou uma alteração dos terços de intersecção do capítulo pela linha umeral anterior em radiografias de perfil.

**Fig. 1:** Radiografias anteroposterior (AP) e de perfil (P) do úmero distal, realizadas imediatamente após a cirurgia, que demonstram redução insatisfatória, visto que a linha umeral anterior não toca o capítulo. Na incidência AP, o fio da coluna ulnar parece não estar fixando o fragmento distal.

Também foram avaliadas as variáveis de configuração da fixação: organização dos fios e estabilidade promovida (número de corticais, local de cruzamento, divergência dos fios). A técnica de fixação foi considerada inadequada quando existia: 1) falta de fixação cortical do fragmento distal ou proximal por um ou mais fios, num total de menos de quatro corticais fixadas; 2) convergência dos fios na cortical proximal; ou 3) pinos se cruzando no foco da fratura 10 ( Fig. 2 ). A fixação das quatro corticais, duas no fragmento proximal e duas no distal, com separação adequada para fixar duas colunas, foi considerada boa para o propósito deste estudo. Os períodos do dia em que a cirurgia foi realizada foram estratificados em diurno (7–19h), noturno (19–00h) ou madrugada (00–7h).

**Fig. 2:** Radiografias AP, P e oblíqua do úmero distal, realizadas imediatamente após a cirurgia, que demonstram uma redução aceitável mas uma fixação inadequada, visto que um dos fios não está fixando o fragmento distal.

Os dados foram coletados mediante revisão de prontuário e avaliados por testes estatísticos específicos (teste do Qui-quadrado) pelo aplicativo do programa IBM SPSS Statistics for Windows (IBM Corp., Armonk, NY, Estados Unidos), versão 25.0, com um nível de significância de 0,05. O projeto foi aprovado de acordo com a Resolução no 466/2012 do Conselho Nacional de Saúde (CNS) e com as normativas éticas complementares vigentes.

## Resultados

Ao todo, foram avaliados 194 casos de fraturas supracondilianas submetidas ao tratamento cirúrgico no período estimado. Desses pacientes, 133 eram do sexo masculino (68,6%) e 61, do sexo feminino (31,4%). O pico de incidência foi dos 4 aos 7 anos, com uma idade média de 6 anos. O lado mais acometido foi o esquerdo, em 57,8% dos casos. Em relação ao desvio da fratura, 171 delas foram em extensão (87,6%), e 24, em flexão (12,4%).

Lesão neurológica foi documentada em 13 casos (6,7%), sendo que em 5 pacientes houve o acometimento do nervo ulnar, em 4, a lesão do mediano, em 3, a lesão do nervo radial e, em 1 paciente, lesão do nervo radial associada a comprometimento do nervo mediano. Todos os casos obtiveram recuperação completa da lesão neurológica em um período de 6 meses. A complicação mais grave foi a síndrome compartimental, que aconteceu em 1 caso, que necessitou de fasciotomia, mas evoluiu para contratura isquêmica de Volkmann. Em relação à abordagem, 4 casos (2,1%) foram reduzidos de forma aberta, sendo os demais realizados mediante redução indireta e fixação percutânea.

As fraturas foram adicionalmente classificadas pelo traço: 173 foram classificadas como transversa baixa, 7, como oblíqua medial, 6, como oblíqua lateral, e 8, como transversa alta. Das fraturas transversas baixas, 42 apresentaram traço alto no plano sagital, e foram consideradas instáveis. No total, 32,47% (63 casos) das fraturas foram consideradas instáveis por sua morfologia.

A fixação com fios cruzados foi usada em 125 casos (64,5%), com 2 fios laterais, em 35 casos (18%), com 2 fios laterais e 1 fio medial, em 27 casos (14%), com 3s fios laterais, em 6 casos (3%), e houve 1 caso com configuração atípica, com 1 fio lateral e 3 fios mediais (0,5%). Do total, 55 casos (28,35%) apresentaram fixação insuficiente.

A redução pós-operatória foi considerada aceitável em 161 pacientes (82,9%). Ao todo, 33 fraturas (17%) foram fixadas em parâmetros fora do ideal em termos do ângulo de Baumann e da linha umeral anterior, sendo 5 desses submetidos a reintervenção. O principal indicativo de má redução ocorreu no plano sagital, quando a linha umeral anterior não encontrou nem mesmo o terço anterior do capítulo. Das fraturas mal reduzidas, apenas 1 foi do tipo II de Gartland, sendo as demais do tipo III ( *p* = 0,042). Em relação à instabilidade do traço da fratura, das 33 fraturas instáveis, 15 (45,45%) obtiveram uma redução inadequada constatada no pós-operatório imediato ( *p* = 0,105).

Em relação ao horário da cirurgia, 19 pacientes (25,7%) dos atendidos no turno noturno ou da madrugada tiveram redução insatisfatória, comparados a 14 (11,7%) dos operados no período diurno ( *p* = 0,020).

A perda de redução aconteceu em 39 casos (20,10%), sendo 37 destes em fraturas do tipo III de Gartland ( *p* = 0,032), e 16 (42%), em fraturas morfologicamente instáveis ( *p* = 0,174). As perdas de redução ocorreram no plano coronal (ângulo de Baumann) em 24 pacientes, no plano sagital (linha umeral anterior) em 8, e em ambos os planos em 7 casos ( Figs. 3 e 4 ). De todos os pacientes que perderam redução, 19 (48,7%) apresentavam fixação insuficiente ( *p* = 0,002). Por fim, a perda de redução totalizou 15 casos (12,5%) dos operados durante o dia, comparados a 24 (32%) dos operados no período noturno ou da madrugada ( *p* = 0,001).

**Fig. 3:** Radiografias AP e P do úmero distal realizadas imediatamente após a cirurgia.

**Fig. 4:** Radiografias AP e P do mesmo paciente da fig. 3 três semanas após a fixação, demonstrando a perda da redução.

## Discussão

A epidemiologia quanto à idade dos pacientes com fraturas supracondilianas do úmero é concordante na maioria das referências, sendo mais frequente na faixa etária entre 4 e 7 anos. 3 4 As fraturas em extensão prevaleceram na amostra deste estudo; contudo, houve alta incidência de desvios em flexão (12,4%) quando comparada à da literatura mundial (2%). 8

A neuropraxia isolada do nervo interósseo anterior (NIA) é a lesão nervosa mais frequente nos desvios em extensão, 11 ao passo que o ulnar é acometido em 90% das fraturas em flexão. 12 Estima-se que a lesão iatrogênica do ulnar ocorra em 1 a cada 28 pacientes (4%) quando utilizada fixação cruzada. 13 Portanto, a incidência aumentada de 12,4% de acometimento ulnar na amostra deste estudo pode estar relacionada à maior incidência de fraturas em flexão, associada à ampla utilização de fixação cruzada. A complicação mais grave foi a síndrome compartimental, ocorrida somente em 1 caso (0,51%), semelhante aos dados de Omid et al., 4 cuja incidência foi de 0,1 a 0,5%. A baixa incidência de complicações relacionadas ao tempo transcorrido entre a lesão e a fixação corrobora o manejo cirúrgico planejado e com segurança, sem necessidade de abordagem como urgência-emergência para a maior parte dos casos.

A maioria das fraturas foram resolvidas mediante redução indireta e fixação percutânea. A necessidade de redução aberta é rara, e gira em torno de 6% dos casos. 8 Neste estudo, a redução aberta foi utilizada 4 vezes (2,1%). As principais indicações são as fraturas irredutíveis, expostas, ou quando há diminuição da perfusão após a redução. 14

A perda de redução após a fixação das fraturas supracondilianas varia de 1,6 a 33,3%. 15 Segundo Skaggs e Flynn, 14 a principal causa disso é a fixação inadequada, que é basicamente atribuída a erros técnicos. Os pinos de entrada lateral devem ser divergentes, buscando o espaçamento máximo no foco da fratura. 16 Não devem convergir e nem se cruzar no foco da fratura; a fixação bicortical é fundamental. 11 Neste estudo, a fixação insuficiente esteve presente em 48,7% das perdas de redução ( *p* = 0,002), e foi fator causal importante de resultados insatisfatórios. A grande heterogeneidade de cirurgiões plantonistas e o fato de que se tratar de um serviço de formação de novos traumatologistas são fatores relevantes nesse contexto.

O horário da cirurgia se mostrou variável importante no desfecho radiológico: 32% dos casos operados à noite ou de madrugada tiveram radiografias insatisfatórias ao final do seguimento. A busca por uma justificativa causal para esse dado envolve o fato de o cirurgião estar fora de seu contexto de maior conforto, em terceiro turno de trabalho e com equipe assistente reduzida. A capacidade técnica ou o treinamento do cirurgião não devem ser fatores determinantes, uma vez que as cirurgias noturnas são realizadas pelo mesmo grupo de cirurgiões das diurnas. Isso não pode ser atribuído exclusivamente ao cirurgião ortopédico, visto que aproximadamente 30 plantonistas realizam plantão diurno e noturno, e fazem a mesma cirurgia em diferentes horários, independentemente da sua subespecialidade. O tratamento cirúrgico durante a noite está associado a uma maior taxa de fixação inadequada quando comparado aos procedimentos diurnos. 17 O retardo da cirurgia para o dia seguinte não aumenta o número de complicações, desde que respeitados os sinais de alarme, como exposição, lesão vascular e sinais de complexo secundário (edema volumoso, equimoses, pinçamentos de tecidos moles). 18

Algumas limitações desta análise precisam ser consideradas: inicialmente, o caráter retrospectivo, com levantamento de dados conforme a disponibilidade em prontuário. Além disso, é fundamental o estudo adicional da correlação clínica dos achados radiológicos aqui descritos.

## Conclusão

A qualidade da redução cirúrgica e sua manutenção no seguimento pós-operatório mostraram-se intimamente relacionadas a aspectos técnicos e ao período do dia em que a cirurgia foi realizada. Portanto, excetuando situações pontuais de maior risco, o retardo da cirurgia para o dia seguinte repercute em melhor desfecho radiológico, sem aumentar a incidência de complicações.
